# Neck circumference as a risk factor of screen-detected diabetes mellitus: community-based study

**DOI:** 10.1186/s13098-016-0129-5

**Published:** 2016-02-16

**Authors:** Mykolay Khalangot, Vitaliy Gurianov, Nadia Okhrimenko, Igor Luzanchuk, Victor Kravchenko

**Affiliations:** Shupyk National Medical Academy of Postgraduate Education, Kiev, Ukraine; Komisarenko Institute of Endocrinology and Metabolism, Vyshgorodska 69, Kiev, 04114 Ukraine; Bohomolets National Medical University, Kiev, Ukraine

**Keywords:** Neck circumference, Screen-detected diabetes mellitus, Thyroid gland volume, Body mass index, Waist to hip index, Risk factor

## Abstract

**Background:**

Whereas an increase of neck circumference (NC) had been recently identified as a new independent cardiovascular disease (CVD) and metabolic syndrome risk factor, similar assessments concerning screen-detected diabetes mellitus (SDDM) have not been made. Thyroid gland volume (ThV) can potentially affect NC however the significance of this influence concerning the risk of NC-related disease is unknown.

**Methods:**

We performed a ThV-adjusted evaluation of NC within a population-based investigation of SDDM and impaired glucose regulation (IGR) prevalence. This study contains fasting plasma glucose (FPG) and 75 g 2-h glucose tolerance test results (2-hPG) of 196 residents of Kyiv region, Ukraine, randomly selected from the rural population older than 44 y.o. who were not registered as diabetes mellitus patients. Standard anthropometric (height; weight; blood pressure; waist, hip circumferences), NC and ultrasonography ThV measurements were performed, hypotensive medication, CVD events and early life nutrition history considered. HbA1c was measured, if FPG/2-hPG reached 7.0/11.1 mmol/l respectively; HbA1c level 6.5 % was considered to be SDDM diagnostic; IGR if FPG/2-hPG reached 6.1/7.8 but less than 7.0/11.1 mmol/l respectively.

**Results:**

Neck circumference among women with normal FPG/2-hPG was 35 (33–36) cm, IGR 36 (34.5–38) cm, SDDM HbA1c < 6.5 % 42 (40–43) cm, HbA1c > 6.5 % 42.5 (40–44) cm, p < 0.001, and for men from the same groups 38.5 (36.5–41.5) cm; 39 (37–42) cm; 42 (40–43) cm; 42.5 (40–44) cm, p = 0.063; medians (Q_I_–Q_III_). Gender-adjusted logistic regression OR for SDDM HbA1c > 6.5 % vs. normal FPG/2-hPG category depending of NC as a continued variable, equaled to 1.60 (95 % CI 1.27–2.02) per cm. Additional adjusting by ThV, body mass or waist/hip index, high blood pressure, acute CVD events, or starvation history did not significantly influence this risk.

**Conclusion:**

Neck circumference is a new risk factor of SDDM that is independent from other indicators of adipose tissue distribution as well as from the ThV.

**Electronic supplementary material:**

The online version of this article (doi:10.1186/s13098-016-0129-5) contains supplementary material, which is available to authorized users.

## Background

The importance of different fat deposition types, as risk factors for developing metabolic disorders and atherosclerosis was established in 1956 by Jean Vague, however the measurements of corresponding circumferences and their quantitative assessment were not performed [1].

Later in 1984, Arthur Hartz, David Rupley, and Alfred Rimm revealed that increasing of the waist circumference (WC) to hip circumference (HC) ratio (WC/HC) was positively associated with having diabetes and/or arterial hypertension or gall bladder disorders, based on questionnaire data of 21,056 women aged 40–59, who were members of a weight-loss program [2]. Shortly after, questionnaire data of a similar, but twice larger group of women, obtained by Freedman and Rimm [3] revealed that prevalence of diabetes mellitus is positively associated with neck circumference (NC). The presence of goiter, quantitative parameters of thyroid gland, or lifestyle characteristics were not taken into account in this study [3].

Strangely, unlike WC/HC ratio that has long been a routine test to assess the risk of type 2 diabetes mellitus (T2D) and cardiovascular disease (CVD) development [4, 5], an increase of NC is not known as a T2D risk factor. Just recently it was shown that NC is a predictor of metabolic syndrome in short-sleeping obese men and women and even addition of NC to the definition of metabolic syndrome was proposed for consideration [6]. Whereas an increase of NC has been recently determined as a new independent risk factor for CVD and metabolic syndrome [7–9], we are not aware of any similar cross-sectional assessments concerning T2D risk. When our manuscript was already being reviewed, results of a prospective, community-based cohort study of Korean adults were published. These results indicate a negative impact from large neck circumference in the development of diabetes mellitus [10].

Cross-sectional risk assessment of NC increase is not provided in the Korean study, and some potentially substantial adjusting, for such factors as thyroid volume or early development conditions was not performed. The issue of the significance of this new anthropometric indicator for assessing risks for screen-detected diabetes mellitus (SDDM) during population-based studies in Europe remains open.

We performed a NC assessment, adjusted by thyroid size, within a recent (2013) screen-based investigation of SDDM (which is usually T2D) and impaired glucose regulation (IGR) epidemiology in rural Ukraine [11]. Ethnic as well as other territorial factors, including early development circumstances can affect the feasibility of risk factors and T2D frequency [12–14]. Therefore an evaluation of T2D risk factors in Ukraine, where the population suffered from mass starvation several times during the twentieth century [15] becomes even more significant, as the estimates of population decline due to the famine range from 2.7 to 3.9 million [16–18].

## Methods

Diabetes and IGR (pre-diabetes) screening was conducted between Jul 2013 and Nov 2014. In total, 202 rural residents over 44 years of age, not registered as T2D patients, were randomly selected. Relevant lists of residents from two towns, provided by family doctors were used for randomization. Patients were selected using random number tables [19], and received an invitation to take part in the study. If the patients did not give consent to take part, the invitation was forwarded to the next person in the randomized list. This study contains test results of 196 residents of Andriivka and Kopyliv villages (Kyiv region), who permanently live in the above communities and signed the informed consent form. All those involved, including the district endocrinologist and family physicians took part in the study as volunteers. The study protocol was approved by the ethics committee of the Institute of Endocrinology and Metabolism, National Academy of Medical Sciences, Ukraine. After signing the informed consent forms the participants filled out questionnaires, providing information about current treatment and lifestyle, as well as about the fact of starvation in their family in 1930s and/or 1946. One hundred and fifty nine persons answered the question about starvation of parental family, including 73 born before 1947. Among them 62 persons answered positive about starvation of parental family. Sufficient physical activity level (30 min/day) was determined in accordance with current T2D prevention guidelines [20].

Anthropometric measurements and glucose tolerance tests were performed. Glicosylated hemoglobin levels (HbA1c) was measured, if fasting/2 h plasma glucose reached 7.0/11.1 mmol/l respectively; HbA1c level 6.5 % was considered to be diabetes mellitus diagnostic. IGR (prediabetes) was considered in case if fasting plasma glucose reached 6.1/7.8 mmol/l but was below 7.0/11.1 mmol/l respectively.

### Anthropometric measurements

Body mass was measured using well-tried electronic scales, height—using standard portable stadiometer. Waist circumference (WC), hip circumference (HC), and neck circumference (NC) were measured with a flexible measuring tape at maximum transverse size in standing position. NC was measured above the cricothyroid cartilage to 5 mm accuracy.

Body mass index (BMI) was determined as a relation of body mass in kilograms to squared height in meters. To measure arterial blood pressure (BP) we assessed the Korotkoff sounds using operational blood pressure monitors from corresponding family medicine clinics. BP was measured twice, with an interval of 5 min. If there was a difference of more than 10 mm we made a third measurement. The mean value of these two/three measurements was counted. High BP was determined as 140/90 mmHg and above, or by the fact of hypotensive drug treatment. The blood sampling was done after at least 10 h of fasting and 2 h after taking a glucose solution (75 g of glucose in 200 ml of water). Blood plasma was quickly separated with a centrifuge and stored in a cold environment for further testing during 24 h. Glucose and HbA1c levels were determined by standard methods and in a certified lab: glucose oxidase method was used for glucose testing. HbA1c levels were assessed using ClOVER A1c (Inforia Co., Ltd.) system that uses boronate resin to bind HbA1c.

Thyroid ultrasonography studies were performed and interpreted by the same experienced radiologist (IL), using the same equipment with a linear probe (Terason Ultrasound, Burlington, MA). The subjects were examined in supine position with the neck extended. Thyroid volume was measured as reported previously [21]. Longitudinal and transverse scans were performed, allowing the measurement of the depth, the width and the length of each lobe. The volume of each lobe was calculated by the ellipsoid formula (volume in ml = 0.479 × maximum thickness × maximum length × maximum width). Total thyroid volume was the sum of both lobes, and the isthmus was not included. The diagnosis of hyperthyroidism was not previously known in any of the studied persons. In all cases determining thyroid volume, when it was increased or there were nodes over 2 cm in diameter, one of the authors (MKH) assessed the signs and symptoms of hyperthyroidism and recommended measuring TSH and T4. Hyperthyroidism was not found in any of the studied individuals.

### Statistical analysis

In order to evaluate the distribution of qualitative indicators we calculated the manifestation frequency (%), whereas quantitative indicators, due to their nonparametric distribution in many cases, were given as medians and 1–3 quartiles. Frequency of events was compared using Chi squared (Yates corrected). If at least one row of quantitative data had a non-normal distribution, then Kruskal–Wallis test was used, else we used ANOVA for comparisons of four groups. We also evaluated odds ratios (ORs) and corresponding 95 % confidence intervals (CIs) in order to assess the risk of events in cross-sectional studies using the model of logistic regression. ROC analysis was performed to assess the accuracy of models and to determine optimal cut-off thresholds. Youden index (sensitivity + specificity − 1) was used for determining the cut-off point. SPSS 11.0 and MedCalc v. 15.6 (MedCalc Software Inc., Broekstraat, Belgium) software packages were used for statistical analysis. All tests of significance were two tailed with the critical level of p = 0.05.

## Results

From 196 men and women, 74 (37.8 %) demonstrated normal fasting glucose and glucose tolerance (NGT), and 54 (27.6 %) had diabetic glucose levels in venous plasma (SDDM). Among them there were 25 persons (12.7 %), whose HbA1c level reached 6.5 %, hence were considered having chronic hyperglycemia—confirmed diabetes mellitus. Members of this group had higher, comparing to others, levels of plasma glucose, had a more frequent history of strokes and/or myocardial infarctions (p < 0.05) and all had arterial hypertension. The fraction of those, who starved during childhood in this group was smaller (p = 0.002), comparing to others. Sixty eight persons (34.7 %), according to glucose tolerance testing were assigned to impaired glucose regulation (IGR) category or pre-diabetes. Distribution of these four categories (NGT; IGR; SDDM HbA1c < 6.5 %/HbA1c ≥ 6.5 %) among women and men was the same. Among anthropometric indicators, increasing of glycaemia from NGT category to HbA1c ≥ 6.5 % in women is associated with elevation of BMI, WC, HC, WC/HC, NC (p < 0.05). For men this is true (p < 0.05) only concerning WC and WC/HC ratio (Table 1).

**Table 1:** Anthropometric and life style characteristics of rural residents (45+ years old, Kyiv region, Ukraine) belonging to different categories, created according to a results of the glucose tolerance screening

Categories/measurements	NGT	IGR: IFG and/or IGT	SDDM, Hb A1c < 6.5 %	SDDM, Hb A1c ≥ 6.5 %	*P*
Both genders, n	74	68	29	25	
Fasting glucose, mmol/l	5.6 (5.2–5.8)	6.3 (6.1–6.6)	7.4 (7.1–7.9)	8.1 (7.4–9.4)	<0.001
Glucose tolerance test, mmol/l	5.5 (4.8–6.7)	6.8 (5.5–8.9)	7.9 (6.4–11.5)	12.6 (8.4–15.6)	<0.001
HbA1c, %; n	5.25 (5.01–5.5); 2	5.8 (5.6–5.8); 14	5.9 (5.6–6.2); 29	7.3 (7.0–8.0); 25	<0.001
Insufficient physical activity, n (%)	6 (8.2)	4 (5.9)	6 (20.7)	5 (20.0)	0.061
First line relatives with diabetes, n (%)	14 (19.2)	14 (20.9)	6 (22.2)	4 (17.4)	0.970
Current smoking, n (%)	6 (8.1)	4 (5.9)	2 (6.9)	1 (4.0)	0.895
Alcohol consumption, n (%)	50 (68.5)	37 (54.4)	29 (62.1)	15 (60.0)	0.395
Stroke/MI history, n (%)	5 (6.8)	3 (4.5)	5 (17.2)	19 (76.0)	<0.001
High blood pressure, n (%)	48 (64.9)	49 (72.1)	23 (79.3)	25 (100)	0.006
Parental family starvation, n (%)	48 (81.4)	44 (83)	16 (76.2)	13 (65)	0.364
Personal childhood starvation, n (%)	26 (89.7)	22 (100)	7 (77.8)	7 (53.8)	0.002
Thyroid volume, ml; n	12.4 (10.6–16.5); 25	13.5 (12.0–17.8); 32	15.7 (14.2–20.6); 12	17.2 (11.5–20.7); 15	0.245
Women, n	55	53	24	18	0.756
Fasting glucose, mmol/l	5.7 (5.3–5.8)	6.4 (6.2–6.6)	7.4 (7.1–7.9)	8.5 (7.4–9.9)	<0.001
Glucose tolerance test, mmol/l	5.7 (5.0–6.8)	7.1 (5.6–8.9)	8.1 (6.6–11.6)	12.5 (8.1–15.7)	<0.001
HbA1c, %; n	–	5.8 (5.6–5.8); 14	5.9 (5.6–6.3); 24	7.5 (7.0–8.0); 18	<0.001
Insufficient physical activity, n (%)	3 (5.5)	2 (3.8)	4 (16.7)	3 (16.7)	0.109
Parental family starvation, n (%)	36 (83.7)	36 (81.8)	12 (75)	12 (80)	0.875
Personal childhood starvation, n (%)	20 (90.9)	18 (100)	5 (71.4)	6 (66.7)	0.045
1st line relatives with diabetes, n (%)	11 (20.4)	12 (23.1)	5 (21.7)	3 (50.0)	0.439
Current smoking, n (%)	1 (1.8)	1 (1.9)	–	–	0.852
Alcohol consumption, n (%)	36 (66.7)	28 (52.8)	15 (62.5)	9 (50.0)	0.412
High blood pressure, n (%)	39 (70.9)	45 (84.9)	20 (83.3)	18 (100)	0.035
Stroke/MI history, n (%)	3 (5.5)	3 (5.8)	4 (16.7)	5 (27.8)	0.021
Age, years	64 (57–74)	63 (56–71)	64 (55–72.5)	68 (60–73)	0.391
Height, cm	158 (154–163)	160 (154–162)	161.5 (155–166)	161.5 (155–164)	0.464
BMI, kg/m^2^	29.7 (25.5–33.3)	32.6 (28.9–36.7)	33.1 (28.8–37.2)	36.6 (31.2–40.4)	0.002
Neck circumference, cm	35 (33–36)	36 (34.5–38)	38 (36–39.3)	37.5 (36–40)	<0.001
Waist circumference, cm	95 (88–103)	105 (98–111)	106 (97.5–114.5)	116 (105–130)	<0.001
Hip circumference, cm	107 (101–117)	109 (102–117)	110.5 (101.5–121)	116 (112–129)	0.008
WC/HC	0.88 (0.83–0.95)	0.93 (0.90–0.98)	0.97 (0.89–1.00)	0.98 (0.95–1.04)	0.014
Thyroid volume, ml; n	12.2 (8.8–15.6); 19	13.5 (12.1–18.1); 25	15.4 (14.6–19.7); 9	15.2 (9.2–22.7); 10	0.173
Men, n	19	15	5	7	
Fasting glucose, mmol/l	5.4 (4.7–5.9)	6.1 (5.6–6.3)	7.4 (7.3–7.4)	7.9 (6.6–9.4)	<0.001
Glucose tolerance test, mmol/l	5.3 (4.3–6.1)	6.1 (5.1–9.1)	6.3 (6.0–7.4)	12.8 (8.4–15.6)	<0.001
HbA1c, %; n	–	–	6.0 (5.6–6.1); 5	7.1 (7.0–8.3); 7	0.003
Insufficient physical activity, n (%)	3 (16.7)	2 (13.3)	2 (40)	2 (28.6)	0.549
Parental family starvation, n (%)	12 (75)	8 (88.9)	4 (80)	1 (20)	0.044
Personal childhood starvation, n (%)	6 (85.7)	4 (100)	2 (100)	1 (25)	0.045
First line relatives with diabetes, n (%)	3 (15.8)	2 (13.3)	1 (25.0)	1 (14.3)	0.953
Current smoking, n (%)	5 (26.3)	3 (20)	2 (40)	1 (14.3)	0.740
Alcohol consumption, n (%)	14 (73.7)	9 (60)	3 (60)	6 (85.7)	0.598
High blood pressure, n (%)	9 (47.4)	4 (26.7)	3 (60)	7 (100)	0.015
Stroke/MI history, n (%)	2 (10.5)	–	1 (20)	1 (14.3)	0.459
Age, years	66 (56–75)	57 (49–78)	61 (59–70)	75 (61–79)	0.364
Height, cm	172 (167–176)	168 (164–172)	167 (167–174)	169 (161–180)	0.294
BMI, kg/m^2^	27.3(24.1–31.1)	28.1 (25.1–33.7)	31.4 (29.0–31.7)	31.9 (29.6–35.9)	0.172
Neck circumference, cm	38.5 (36.5–41.5)	39 (37–42)	42 (40–43)	42.5 (40–44)	0.063
Waist circumference, cm	97 (90–104)	96 (89–109)	108 (99–112)	114 (111–116)	0.029
Hip circumference, cm	100 (96–109)	101.5 (98–108)	97 (97–109)	107 (104–114)	0.315
WC/HC	0.96 (0.93–1.01)	0.97 (0.93–1.02)	1.01 (1.01–1.02)	1.03 (1.01–1.07)	0.026
Thyroid volume, ml; n	14.5 (11.9–28.2); 6	13.5 (11.9–17.5); 7	20.7 (13.9–26.1); 3	17.2 (12.1–20.7); 5	0.655

Data are given as number of persons (percentages) or medians (1, 3 quartiles)

**Fig. 1:**
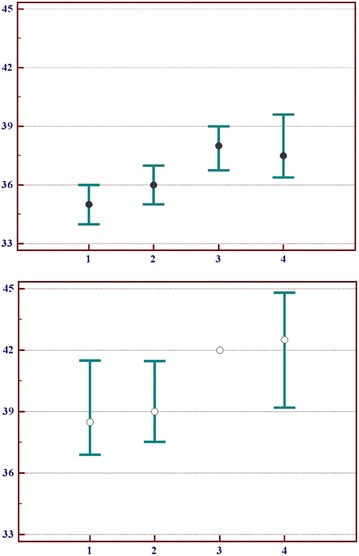
Neck circumference (cm) of rural residents (45+ years old, Kyiv region, Ukraine) belonging to different categories, created according to a results of the glucose tolerance screening. Shown are medians and 95 % CI. *Top panel* (black globes)—women, *low panel* (white globes)—men. 1—NGT; 2—IGR; 3—“Diabetic” glucose levels and Hb A1c < 6.5 %; 4—HbA1c ≥ 6.5 %. 95 % CI for the 3rd male category not given due to insufficient number of persons in this group

We did not find any statistically significant difference of thyroid volume in categories, identified according to the results of glucose tolerance testing, however we did find a correlation of this indicator with NC: according to Spearman’s rank correlation coefficient Ro factor = 0.278; N = 89; p < 0.01. Increase of NC among men and women in categories from normal blood glucose to diabetes mellitus is given in Fig. 1. There are also gender differences that manifest with greater NC among men.

For further analysis of SDDM risk, associated with NC we used ROC curve analysis. 1st category (NTG) and 4th category (HbA1c ≥ 6.5 %) were considered the most distinct concerning the presence or absence of SDDM and they were therefore evaluated separately. Cut-off value concerning NC, chosen using maximization of Youden index for women = 36.5 cm (Table 2).

**Table 2:** Comparison of sensitivity, specificity, youden index of screen-detected T2D (HbA1c ≥ 6.5 %), with different neck circumference point

Neck circumference, cm	SDDM HbA1c ≥ 6.5 % vs. all other study categories			SDDM HbA1c ≥ 6.5 % vs. NGT		
	Sensitivity (%)	Specificity (%)	Youden index	Sensitivity (%)	Specificity (%)	Youden index
Women						
≥29	100.0	0.0	0.00	100.0	0.0	0.00
>31	100.0	6.2	0.06	100.0	13.2	0.13
>32	94.4	10.0	0.04	94.4	18.9	0.13
>33	94.4	18.5	0.13	94.4	26.4	0.21
>34	88.9	29.2	0.18	88.9	39.6	0.29
>34.5	88.9	31.5	0.20	88.9	43.4	0.32
>35	83.3	45.4	0.29	83.3	60.4	0.44
>36	72.2	61.5	0.34	72.2	77.4	0.50
>36.5	72.2	62.3	0.35	72.2	79.3	0.51
>37	50.0	73.1	0.23	50.0	88.7	0.39
>37.5	50.0	74.6	0.25	50.0	90.6	0.41
>38	33.3	82.3	0.16	33.3	90.6	0.24
>38.5	33.3	83.9	0.17	33.3	94.3	0.28
>39	27.8	90.8	0.19	27.8	96.2	0.24
>39.5	27.8	91.5	0.19			0.00
>40	22.2	93.9	0.16	22.2	100.0	0.22
>40.5	16.7	93.9	0.11	–	–	–
>41	5.6	94.6	0.00	–	–	–
>42	0.0	97.7	0.98	0.0	100.0	0.00
>44	0.0	100.0	0.00	–	–	–
Men						
≥35	100.0	0.0	0.00	100.0	0.0	0.00
>38.5	100.0	38.5	0.38	100.0	52.6	0.53
>39	83.3	51.3	0.35	83.3	52.6	0.36
>40	66.7	66.7	0.33	66.7	68.4	0.35
>41.5	66.7	71.8	0.38	66.7	79.0	0.46
>42	50.0	84.6	0.35	50.0	89.5	0.39
>43	33.3	89.7	0.23	33.3	89.5	0.23
>44	16.7	100.0	0.17	16.7	100.0	0.17
>45	0.0	100.0	0.00	0.0	100.0	0.00

Exceeding of this NC value indicated a higher statistical possibility of falling into SDDM HbA1c ≥ 6.5 % category, and not into others, identified according to glycaemia and glucose tolerance screening results (area under curve-AUC = 0.690 ± 0.064, p = 0.003) with sensitivity 72.2 % (95 % CI 46.5–90.3 %) and specificity 62.3 % (95 % CI 53.4–70.7 %). If we were to compare only SDDM HbA1c ≥ 6.5 % and NTG categories, the corresponding ROC values that indicate statistical significance of the test will only increase (AUC = 0.800 ± 0.063, p < 0.0001) sensitivity 72.2 % (95 % CI 46.5–90.3 %) and specificity 79.3 % (95 % CI 65.9–89.2 %). Odds ratios (ORs) of diagnosing SDDM HbA1c ≥ 6.5 % for these two models, providing the use of NC cut off point > 36.5 cm are 4.3 (95 % CI 1.4–12.8), p = 0.009 or 9.9 (95 % CI 2.9–33.8), p < 0.001 respectively. The use of male NC cut-off > 38.5 cm in other statistical models has also proven its predictive value concerning SDDM diagnostics: AUC = 0.774 ± 0.092, p = 0.003 and 0.803 ± 0.095, p = 0.003 (p value comparing to AUC = 0.5) in case of comparing HbA1c ≥ 6.5 % to all other categories, or just to NTG. Among men, the indicated models demonstrated 100 % (95 % CI 54, 1–100 %) sensitivity, but low specificity: 38.5 % (95 % CI 23.4–55.4 %) and 52.6 % (95 % CI 28.9–75.6 %). Assessment according to logistic regression model of gender-adjusted chances of falling into T2D HbA1c ≥ 6.5 % versus NTG categories that are associated with an increase of NC, indicates a preservation of their statistical significance, and sometimes its increase after additional adjusting by personal history (models #1, 2) or anthropometric characteristics (models #3–6); see Table 3.

**Table 3:** Logistic regression multivariable evaluating of SDDM HbA1c ≥ 6.5 % vs. NGT category neck circumference risk

	Models # and their adjusting	OR (95 % CI)	P
0	Gender	1.60 (1.27–2.02)	<0.001
1	Gender + stroke/MI history	1.59 (1.26–2.01)	<0.001
2	Gender + personal childhood starvation	1.92 (1.25–2.97)	0.003
3	Gender + BMI	1.43 (1.05–1.96)	0.024
4	Gender + WC/HC	1.53 (1.21–1.94)	<0.001
5	Gender + high blood pressure	1.57 (1.21–2.03)	<0.001
6	Gender + thyroid volume	2.01 (1.20–3.38)	0.008

OR’s calculated for the NC unit (cm) as continued variable

Comparing corresponding areas under curves (AUCs), built according to models 0–6 indicates that there are no differences between models #1–6 and model #0 (Additional file 1: Fig. S1; Additional file 2: Fig. S2).

## Discussion

Thus, in a relatively small (n = 196) cross-sectional study, recently conducted among residents of rural Ukraine, aged over 44 y.o., based on standard glucose tolerance testing, we found a substantial fraction of persons (27.6 %) with diabetic plasma glucose levels, and 12.7 % were diagnosed with diabetes (confirmed by HbA1c ≥ 6.5 %). 37.8 % of persons demonstrated normal fasting glucose, along with normal glucose tolerance, whereas 34.7 % were identified as prediabetic, according to glucose tolerance testing. Unfortunately Ukraine had no epidemiologic studies, involving prevalence of T2D/IgR or impaired glucose tolerance after introduction of current WHO (1999) blood glucose diabetes/IGR criteria, therefore we could only use data from other countries for comparison. According to recent epidemiologic data from International Diabetes Federation [22], worldwide prevalence of diabetes mellitus grows from around 11 % at 45 y.o. to 20 % at 75 y.o. A very large Chinese study [23], the design of which (oral glucose-tolerance test performed in randomly selected and population-based cohort) was similar to ours, showed a prevalence of 20.4 % for those aged 60 and above [23].

Considering the fact that in the four categories that we identified according to glucose tolerance testing, the age medians fluctuated from 57 to 75 years for men and from 63 to 68 years for women, the quantitative glucose tolerance test-based distribution of persons among the categories does not look impossible or improbable. Considering a small scale of our study, as well as some practical considerations, we have isolated the category of persons with “diabetic” fasting and/or standard glucose loading levels and HbA1c ≥ 6.5 %. In other words, chronic hyperglycemia (diabetes mellitus) in this group (group 4) was better confirmed, than in group 3 (“Diabetic” glucose levels, HbA1c < 6.5 %). Group 4 was later used to build statistical models to assess the chances of detecting diabetes during screening that were associated with questionnaire or anthropometric data. As far as we know, such approach to classification of subjects was not previously used.

Thus, our classification (1—NGT; 2—IGR; 3—“Diabetic” glucose levels and HbA1c < 6.5 %; 4—HbA1c ≥ 6.5 %), despite a relatively small number of persons, is confirmed by results of biochemical tests (higher levels of plasma glucose in group HbA1c ≥ 6.5 % vs. HbA1c < 6.5 %), anthropometric measurements (BMI, WC, HC, HC, WC/HC, NC), as well as questionnaire data/BP measurements (stroke/MI history; high BP; personal childhood starvation). A positive relation of most of these factors with T2D risk is well known [24, 25]. Association of NC or childhood starvation with SDDM risk could be considered new data. The latter factor deserves to be analyzed separately therefore in this study it was considered only as one of the causes that could potentially influence the correlation between NC and diabetes risk.

We believe that an increase of NC in men and women starting from normal plasma glucose group and ending with diabetes mellitus group that is statistically significant for women (<0.001), and is a tendency for men (Table 1; Fig. 1), are the most interesting results of this study. According to Receiver Operating Characteristics (ROC) analysis, the statistically significant NC cut-off points for women and men were 36.5 and 38.5 accordingly, and reaching/exceeding these values would indicate a statistically significant risk of having SDDM. Recently a team of researchers from China [9] confirmed that NC ≥ 37 cm for men and ≥ 33 for women were the best cut-off points for metabolic syndrome, however the presence of diabetes mellitus was not determined in this study [9]. Using the obtained in our study NC cut-off point for women as a categorical variable allowed to evaluate the corresponding OR = 9.9 (95 % CI 2.9–33.8).

Performed chance assessment of finding screen-detected diabetes, associated with an increase of NC by univariate and multivariate logistic regression analyses in models that were adjusted for gender and anthropometric (BMI, WC/HC, thyroid volume) or other (stroke/MI; childhood starvation history; high BP) confounders, confirmed independent nature of this risk factor. Gender-adjusted OR, determined by an increase of NC as a continued variable equaled to 1.60 (1.27–2.02). As far as we know, there were no previous risk assessments for screen-detected T2D, associated with NC. In a cross-sectional study of 43,595 women participating in the Take Off Pounds Sensibly Club, those with a self-reported neck circumference in the top tertile were found to have a twofold increased risk of diabetes relative to those in the bottom tertile, even after adjustment for other measures of adiposity (BMI, WC/HC) [3]. Our study is population-based and is set up on our own measurements that included glucose tolerance testing, anthropometric data, and thyroid volume.

Studies, that assessed the significance of neck circumference in the context of cardiometabolic risk indicate that NC is associated with CVD risk factors even after adjustment for multidetector computed tomography assessed visceral adipose tissue [7] and measured by dual-energy X-ray absorptiometry head fat was positively corrected with NC in males but not females. There was no significant correlation between head fat and fasting plasma glucose [26].

In prospective, community-based cohort study of Korean adults, it was found that the highest quartile of neck circumference was associated with a 1.746- and 2.077-fold higher risk of DM development in men and women respectively, after adjusting for various factors that are known to affect glucose metabolism. [10].

In our study we managed to show SDDM risk, in a cross-sectional population-based study of adults in rural Ukraine, adjusted by ThV and childhood starvation history.

One of the more evident explanations of association between NC and diabetes risk could be sleep disorders and/or obstructive sleep apnea (OSA), associated with an increase of NC, as it was recently demonstrated that OSA is a predictor of abnormal glucose metabolism in chronically sleep deprived obese adults [6], a high NC is a predictor of metabolic syndrome and obstructive sleep apnea in short-sleeping obese men and women [27]. However in a study of Cho et al. [10], daytime sleepiness and snoring habit were not associated with incidence of SDDM and did not change the association of NC with SDDM [10].

Furthermore, hypoxia exposure has been shown to exert beneficial effects on glucose homeostasis and insulin sensitivity in humans, but underlying mechanisms have not yet been studied in detail [28].

## Conclusion

Neck circumference measurement during screen-based investigation of glycaemia and glucose tolerance led to unveiling of a new risk factor of screen-detected diabetes, which is independent from other indicators of adipose tissue distribution as well as from the thyroid volume. Future studies are needed to understand the nature of this connection and to better assist the diabetes prevention possibilities.

## Additional files

10.1186/s13098-016-0129-5 Comparing AUCs of the ROC models #0–5 evaluating of screen-detected T2D HbA1c ≥ 6.5 % vs. NGT category neck circumference risk.
10.1186/s13098-016-0129-5 Comparing AUC’s of the ROC models #0 and 6 evaluating of SDDM HbA1c ≥ 6.5 % vs. NGT category neck circumference risk.

## Abbreviations

NC: neck circumference
CVD: cardiovascular disease
SDDM: screen-detected diabetes mellitus
T2D: type 2 diabetes mellitus
NGT: normal fasting glucose and glucose tolerance
ThV: thyroid gland volume
IGR: impaired glucose regulation
FPG: fasting plasma glucose
2-hPG: 75g 2-h glucose tolerance test
WC: waist circumference
HC: hip circumference
WC/HC: waist circumference to hip circumference ratio
HbA1c: glicosylated hemoglobin levels
BMI: body mass index
BP: arterial blood pressure
AUC: area under curve
