# Supplementary material for: Neck circumference as a risk factor of screen-detected diabetes mellitus: community-based study
Source: Diabetol Metab Syndr. 2016 Feb 16;8:12. doi: 10.1186/s13098-016-0129-5 (PMC4754805; doi:10.1186/s13098-016-0129-5)
Supplement: Supplementary file 1 — 10.1186/s13098-016-0129-5 Comparing AUCs of the ROC models #0–5 evaluating of screen-detected T2D HbA1c ≥ 6.5 % vs. NGT category neck circumference risk. [file 13098_2016_129_MOESM1_ESM.docx]

Additional file 1: Figure S1

Comparing AUCs of the ROC models #0-5 evaluating of screen-detected T2D HbA1c ≥ 6.5% vs. NGT category neck circumference risk


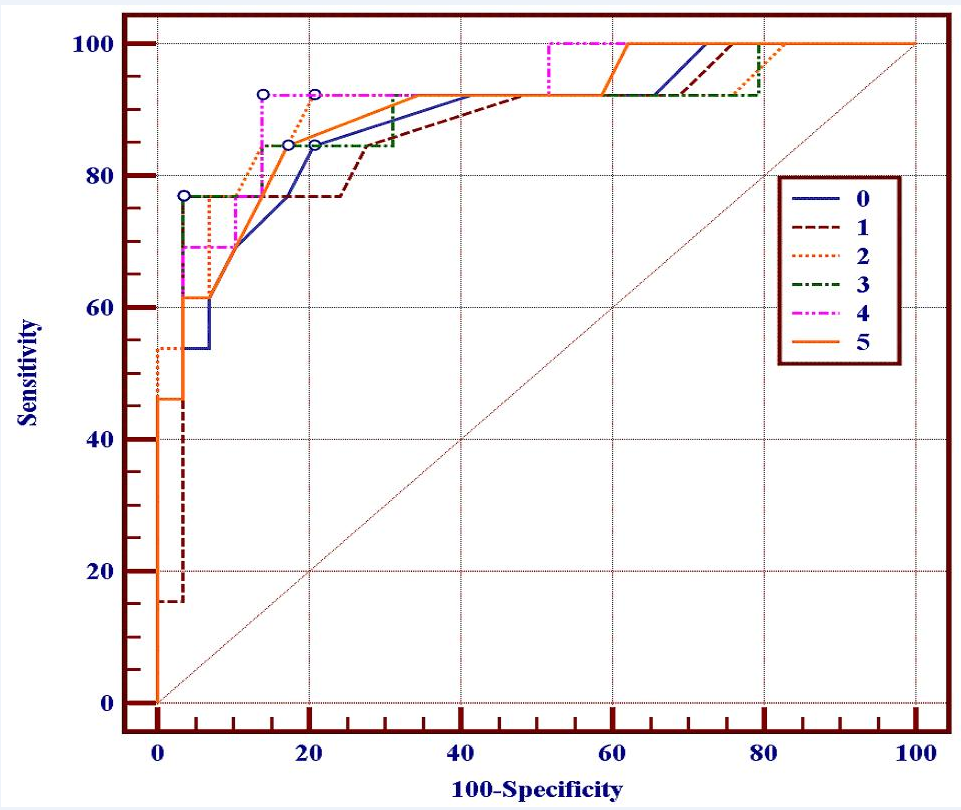


|  | Models # and their adjusting |
| --- | --- |
| 0 | Gender |
| 1 | Gender + Stroke / MI history |
| 2 | Gender +Personal childhood starvation |
| 3 | Gender + BMI |
| 4 | Gender +WC/HC |
| 5 | Gender + High blood pressure |
