# Supplementary material for: Neck circumference as a risk factor of screen-detected diabetes mellitus: community-based study
Source: Diabetol Metab Syndr. 2016 Feb 16;8:12. doi: 10.1186/s13098-016-0129-5 (PMC4754805; doi:10.1186/s13098-016-0129-5)

Additional file 2: Figure S2

Comparing AUC’s of the ROC models # 0 and 6 evaluating of SDDM HbA1c ≥ 6.5% vs. NGT category neck circumference risk

|  | Models # and their adjusting |
| --- | --- |
| 0 | Gender |
| 6 | Gender +Thyroid volume |


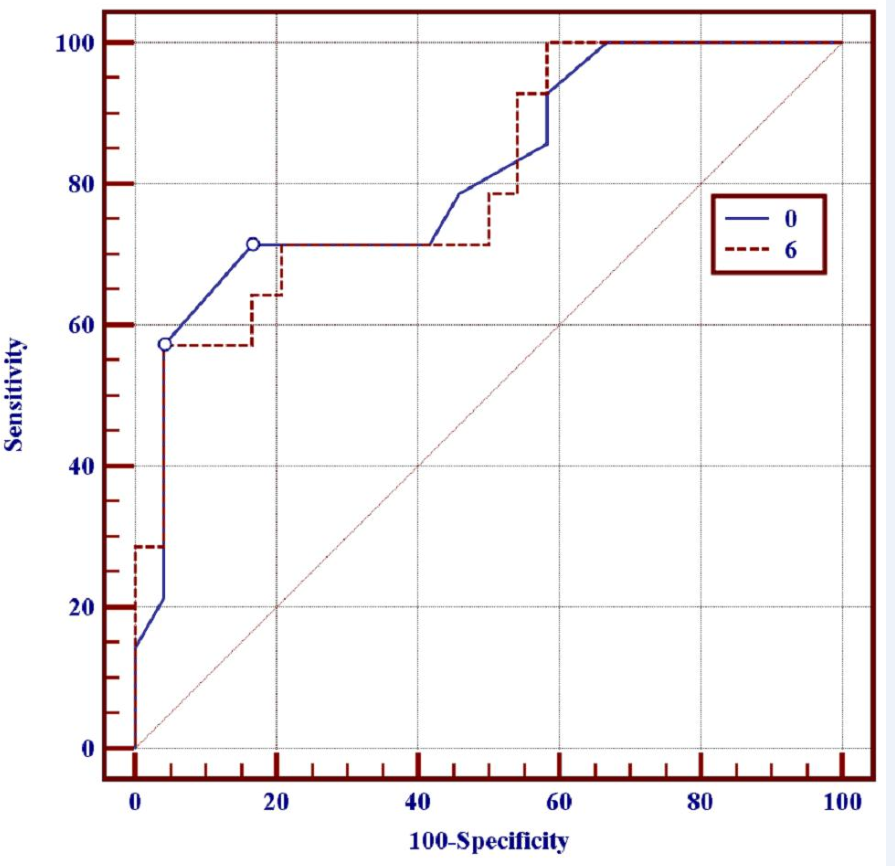

Supplement: Supplementary file 2 — 10.1186/s13098-016-0129-5 Comparing AUC’s of the ROC models #0 and 6 evaluating of SDDM HbA1c ≥ 6.5 % vs. NGT category neck circumference risk. [file 13098_2016_129_MOESM2_ESM.docx]
